# Debate: adjuvant whole brain radiotherapy or not? More data is the wiser choice

**DOI:** 10.1186/s12885-016-2433-8

**Published:** 2016-07-01

**Authors:** Gerald B. Fogarty, Angela Hong, Vinai Gondi, Bryan Burmeister, Kari Jacobsen, Serigne Lo, Elizabeth Paton, Brindha Shivalingam, John F. Thompson

**Affiliations:** Melanoma Institute Australia, Poche Centre, North Sydney, Australia; Sydney Medical School, The University of Sydney, Sydney, Australia; Australia and New Zealand Melanoma Trials Group (ANZMTG), North Sydney, Australia; Trans-Tasman Radiation Oncology Group (TROG), Newcastle, Australia; Princess Alexandra Hospital, Brisbane, Australia; Oslo University Hospital HF, The Norwegian Radium Hospital, Oslo, Norway; Central Dupage Hospital Cancer Center, Warrenville, IL USA; University of Wisconsin Comprehensive Cancer Center, Madison, WI USA; Royal Prince Alfred Hospital, Sydney, Australia; Mater Sydney Radiation Oncology, PO Box 1003, Crows Nest, 1585 Australia

## Abstract

Every year 170,000 patients are diagnosed with brain metastases (BMs) in the United States. Traditionally, adjuvant whole brain radiotherapy (AWBRT) has been offered following local therapy with neurosurgery (NSx) and/or stereotactic radiosurgery (SRS) to BMs. The aim is to increase intracranial control, thereby decreasing symptoms from intracranial progression and a neurological death. There is a rapidly evolving change in the radiation treatment of BMs happening around the world. AWBRT is now being passed over in favour of repeat scanning at regular intervals and more local therapies as more BMs appear radiologically, BMs that may never become symptomatic. This change has happened after the American Society for Radiation Oncology (ASTRO) in Item 5 of its “Choosing Wisely 2014” list recommended: “Don't routinely add adjuvant whole brain radiation therapy to SRS for limited brain metastases”. The guidelines are supposed to be based on the highest evidence to hand at the time. This article debates that the randomised controlled trials (RCTs) published prior to this recommendation consistently showed AWBRT significantly increases intracranial control, and avoids a neurological death, what it is meant to do. It also points out that, despite the enormity of the problem, only 774 patients in total had been randomised over more than three decades. These trials were heterogeneous in many respects. This data can, at best, be regarded as preliminary. In particular, there are no single histology AWBRT trials yet completed. A phase two trial investigating hippocampal avoiding AWBRT (HAWBRT) showed significantly less NCF decline compared to historical controls. We now need more randomised data to confirm the benefit of adjuvant HAWBRT. However, the ASTRO Guideline has particularly impacted accrual to trials investigating this, especially the international ANZMTG 01.07 WBRTMel trial. This is an RCT investigating AWBRT following local treatment in patients with one to three BMs from melanoma. WBRTMel has accrued 196 of a required 220 to date but accrual has slowed. HAWBRT may now never be tested in a randomised setting. Encouraging more data in AWBRT is the wiser choice.

## Background

Brain metastases (BMs) are a significant problem, every year 170,000 patients are diagnosed BMs with in the United States [1]. Traditionally, for oligo metastatic disease (1–4 BMs), adjuvant whole brain radiotherapy (AWBRT) has been offered following local therapy to BMs, which include neurosurgery (NSx) and/or stereotactic radiosurgery (SRS). The aim of the AWBRT is to increase intracranial control, thereby preventing or delaying symptoms from intracranial progression and a neurological death, perhaps even extending survival.

There is a rapidly evolving change in the radiation treatment of BMs happening around the world. AWBRT is now being passed over in favour of repeat scanning at regular intervals and more local therapies as more BMs appear radiologically, BMs that may never become symptomatic.

This change may not be in the best interests of patients, nor of health systems. Patients later in their cancer journey may not be able to access repeat scanning, meaning that some may suffer from intracranial progression and die a neurological death anyway. There may be over treatment with more expensive rescanning and more local therapies like SRS of lesions that were never going to be a problem. All of this is not great palliation nor good use of the health care dollar.

This change has happened after the American Society for Radiation Oncology (ASTRO) in Item 5 of its “Choosing Wisely 2014” list recommended: “Don’t routinely add adjuvant whole brain radiation therapy to SRS for limited brain metastases” [2]. On what basis has this recommendation been made? Is it a truly wise statement?

## Discussion

The randomised controlled trials (RCT) investigating AWBRT published prior to this recommendation are few (Table 1). In these trials, AWBRT has consistently resulted in what it is meant to do. AWBRT significantly increases intracranial control, and avoids a neurological death. There are negative points about these trials. Despite the enormity of the problem, only 774 patients in total have been randomised over more than three decades. The numbers in each trial are small. Two of the five trials, Chang et al. and Roos et al. did not complete accrual, meaning that they are, at best, hypothesis generating. Within each accrual has been slow, often taking over a decade for a reasonably sized trial. The trials are heterogeneous in many respects. The trials included BMs of all histologies of solid malignancies. Most participants had either lung or breast cancer. Patients with lung or breast cancer can have life style characteristics (eg. smoking) and multiple previous systemic chemotherapies that can impact secondary endpoints such as quality of life (QoL) and neurocognitive function (NCF) [3]. The trials differed in the number of BMs allowed, radiotherapy total dose, and dose per fraction, these factors also being important for secondary endpoints. The recent abstract of the Alliance trial presented by Brown at American Society of Clinical Oncology (ASCO) 2015 is a welcome addition to the field. This trial also took over a decade to accrue and comprised BMs of all histologies. The peer – reviewed publication is awaited [4].

**Table 1:** Published Randomised Controlled Trials of AWBRT as of 2015

Study Year of Publication Did trial complete?	Accrual Total /years	Histologies RT dose (Total Gray/#)	No of BMs	Median Overall Survival of all cohort (Months)	Did AWBRT increase intracranial control?	Did AWBRT decrease neurological death?	Adequate NCF Testing
Patchell 1998 [21] Complete	96/8	All 60 % lung 50.4/28	Single	11	Yes	Yes	No
Aoyama 2007 [11, 12] Complete	132/4	All 65 % lung 30/12	1–4	8	Yes	No difference	No
Chang 2009 [14] Incomplete	58/6	All 55 % lung 30/10	1–3	9.2	Yes	No difference	Yes
Roos 2011 [22] Incomplete	19/3	All 30/10	Single	NA	NA	NA	No
Kocher 2011 [23] Complete	359/12	All 50 % lung 30/10	1–3	10.9	Yes	Yes	No

These trials are the only published randomised data on AWBRT that exists. In these trials, AWBRT delivered what it was meant to. This data in total can, at best, be regarded as preliminary. In particular, there are no single histology AWBRT trials yet completed. However, the ASTRO recommendation is opposed to the conclusions of this data.

On what data then is the ASTRO recommendation based? Some SRS enthusiasts have produced a meta-analysis [5, 6], based on the above trials, of 364 selected patients treated with SRS. They conclude that AWBRT should be discouraged as it does not increase overall survival (OS). The findings of the study actually make AWBRT look good. These are tabulated in Table 2 from the figures given in the article. The addition of AWBRT was associated with less local and distant failure, less requirement for local and distant salvage, and less neurological death even in this selected cohort. The addition of AWBRT was also associated with an increase in time to local failure (median from 6.6 to 7.4 months, mean from 11 to 13 months) and time to distant failure (median from 4.7 to 6.5 months and the mean from 9.6 to 12 months). This meta-analysis has already been criticised [7] in the cancer literature. Additional statistical criticisms include that in the article there are no measures of inter-trial consistency reported, as recommended by meta-analysis guidelines [8, 9]. These reports are important as inconsistency of trial homogeneity in a meta-analysis reduces the significance of the findings [10]. The authors also miss the point. AWBRT is essentially a palliative treatment and is primarily about symptom control rather than increasing survival.

**Table 2:** AWBRT details from Sahgal et al. IJROBP 2015 [6]

Parameter	Total Number (364)	Total No AWBRT (186) (SRS only)	Total AWBRT (178)	Impact of addition of AWBRT on parameter
Failure at Local site (as % of totals)	72 (20 %)	51 (27 %)	21 (12 %)	Decreases
Salvage at Local site (as % of fails)	45 (63 %)	37 (73 %)	8 (38 %)	Decreases
Failure of Distant brain(as % of totals)	156 (43 %)	98 (53 %)	58 (34 %)	Decreases
Salvage of Distant brain (as % of fails)	100 (64 %)	72 (73 %)	28 (48 %)	Decreases
Neurologic deaths (as % of totals)	99 (27 %)	55 (30 %)	44 (25 %)	Decreases

An interesting twist is the recent publication by Aoyama et al. [11, 12] of a subgroup re-analysis of 88 patients with non–small cell lung cancer (NSCLC) in the same 2007 Aoyama trial as used in the meta-analysis. Using the disease-specific Graded Prognostic Assessment (ds-GPA), a prognostic stratification tool, the median overall survival time of the favourable group (ds-GPA of 2.5 to 4; 47 patients) was 16.7 versus 10.6 months in the unfavorable group (ds-GPA of 0.5 to 2; 41 patients) [13]. There was a survival advantage in the AWBRT arm over SRS alone (*p* = 0.03) for the favourable group. A similar survival improvement was not observed in the unfavourable group. One possible explanation could be that for patients with a high ds-GPA category, improved brain control allows them to have more systemic therapy and therefore a survival advantage. This sub-study is hypothesis generating, but does confirm the need to consider the histology of the primary cancer and other patient data such as performance status. It also exposes the selection bias and the impact of small numbers in the meta-analysis.

The incompletely accrued Chang trial also showed that AWBRT may be associated with NCF problems in longer term survivors [14]. To address this problem, Gondi et al. have published a phase two trial investigating hippocampal avoiding AWBRT (HAWBRT) [15]. This study showed significantly less NCF decline compared to historical controls. We now need more randomised data to confirm the benefit of adjuvant HAWBRT in intracranial control and NCF preservation.

SRS is also not completely benign. As these patients are living longer, the risk of SRS complications, such as radionecrosis (RN), increases. There may also be a deleterious interaction between SRS and new therapies that may be greater than the interaction with AWBRT alone [16]. Upfront SRS may also risk disappearing in the era of better systemic therapies. A recent trial randomised 105 NSCLC good performance patients with 1–4 BMs, most of them solitary, to either SRS or observation prior to systemic chemotherapy [17]. The median OS time of the whole cohort was 15 months and there was no significant difference between the groups in terms of time to symptomatic progression of BMs, overall central nervous system (CNS) disease progression, or median OS. SRS also did not lead to increased overall survival in this RCT. Should SRS also be dispensed with? Further studies are needed.

The currently accruing international ANZMTG 01.07 WBRTMel trial is investigating AWBRT following local treatment in patients with one to three BMs from melanoma [18]. The ANZMTG 01.07 protocol was first approved by the Cancer Institute NSW Lead Ethics Committee on 20 December 2007 (reference: 2007C/11/032). It is the world’s first single histology AWBRT trial. This trial has all the appropriate NCF measurements and allows HAWBRT. It has accrued 179 of a required 200 to date. Interim analysis has shown the collected data is of high quality [19]. However, trial accrual is decreasing as investigators are not offering the trial because of the ASTRO recommendation. The ASTRO recommendation is stopping much needed data about AWBRT being collected. HAWBRT may never be tested in a randomised setting. ASTRO needs to retract its recommendation.

## Conclusion

BMs are a significant problem. AWBRT is an effective palliative treatment that is based on RCT evidence. Withholding AWBRT leads to a definitive increase in intracranial relapse, not all relapse can be salvaged by Sx or SRS, leads to an Increased cost of observation and managing relapse. It may stop a reduction in neurocognitive decline but the data is weak, and the impact on overall survival is still to be defined. More RCT are needed to improve on AWBRT especially in single histology trials and trials that test HAWBRT. HAWBRT may improve the toxicity profile, especially NCF and needs an RCT to show this.

ASTRO has recently recommended don’t routinely add WBRT to SRS for limited brain metastases as randomized studies have demonstrated no overall survival benefit from the addition of AWBRT to SRS. However, these trials show AWBRT is effective in what it was meant to do and were never powered for OS. Another reason was that the addition of AWBRT to SRS is associated with diminished cognitive function and worse patient-reported fatigue and quality of life, but at the time this was based on an incomplete trial and data sets. A further reason was that careful surveillance and the judicious use of salvage therapy at the time of brain relapse allows appropriate patients to enjoy the highest QoL without a detriment in overall survival but there was no high level of evidence for this. This recommendation is not based on the existing randomised evidence, in fact, the randomised evidence supports the use of AWBRT in oligo metastatic disease. The ASTRO recommendation is impacting accrual to the world’s first single histology trial. AWBRT is being phased out just when radiation oncologists had produced a better radiotherapy technique, HAWBRT, to answer some of these criticisms. ASTRO should consider retracting its recommendation. Encouraging more data in AWBRT is the wiser choice.

The real question to ask is: why are RCT in AWBRT so hard to accrue to when the problem is so common? Studies of poor trial accrual mention many possible factors, but it may be a lack of physician equipoise. This has been found in other cancer types [20]. Lack of equipoise can be driven by many factors, including firmly held views despite little data, fear of losing revenue, and over reliance on phase one and phase two and retrospective data. May the quest for higher level evidence re - invigorate trials in AWBRT.

## Abbreviations

ASCO, American Society of Clinical Oncology; ASTRO, American Society for Radiation Oncology; AWBRT, adjuvant whole brain radiotherapy; BM, brain metastases; CNS, central nervous system; ds-GPA, disease-specific Graded Prognostic Assessment; HA, hippocampal avoiding; NA, not analysed; NCF, neurocognitive function; NSCLC, non–small cell lung cancer; NSx, neurosurgery; OS, overall survival; QoL, quality of life; RCT, randomised controlled trial; RN, radionecrosis; SRS, stereotactic radiosurgery

## References

[1] Platta CS, Khuntia D, Mehta MP (2010). Current treatment strategies for brain metastasis and complications from therapeutic techniques: a review of current literature. Am J Clin Oncol.

[2] ASTRO releases second list of five radiation oncology treatments to question, as part of national Choosing Wisely® campaign. 2014. www.choosingwisely.org/astro-releases-second-list. Accessed 1 July 2016.

[3] Stouten-Kemperman MM, de Ruiter MB, Koppelmans V (2015). Neurotoxicity in breast cancer survivors ≥10 years post-treatment is dependent on treatment type. Brain Imaging Behav.

[4] Brown P, Asher AA, Ballman K, et al. NCCTG N0574 (Alliance): A phase III randomized trial of whole brain radiation therapy (WBRT) in addition to radiosurgery (SRS) in patients with 1 to 3 brain metastases. 2015 ASCO Annual Meeting; 2015. http://meeting.ascopubs.org/cgi/content/short/33/15_suppl/LBA4. Accessed 1 July 2016.

[5] Sahgal A, Larson D, Knisely J (2015). Stereotactic radiosurgery alone for brain metastases. Lancet Oncol.

[6] Sahgal A, Aoyama H, Kocher M (2015). Phase 3 trials of stereotactic radiosurgery with or without whole-brain radiation therapy for 1 to 4 brain metastases: individual patient data meta-analysis. Int J Radiat Oncol Biol Phys.

[7] Arvold ND, Catalano PJ (2015). Local therapies for brain metastases, competing risks, and overall survival. Int J Radiat Oncol Biol Phys.

[8] Moher D, Liberati A, Tetzlaff J, The PRISMA Group, et al. Preferred Reporting Items for Systematic Reviews and Meta-Analyses: The PRISMA Statement. PLoS Med. 6(7):e1000097. doi:10.1371/journal.pmed.1000097. Accessed 1 July 2016. 10.1371/journal.pmed.1000097 PMC2707599 19621072

[9] Higgins JPT, Green S (editors): Cochrane Handbook for Systematic Reviews of Interventions Version 5.1.0 [updated March 2011]. The Cochrane Collaboration, 2011. Available from www.cochrane-handbook.org. Accessed 1 July 2016.

[10] Higgins JPT, Thompson SG, Deeks JJ (2003). Measuring inconsistency in meta-analyses. BMJ.

[11] Aoyama H, Shirato H, Tago M (2006). Stereotactic radiosurgery plus whole-brain radiation therapy vs stereotactic radiosurgery alone for treatment of brain metastases: a randomized controlled trial. JAMA.

[12] Aoyama H, Tago M, Shirato H (2015). Stereotactic radiosurgery with or without whole-brain radiotherapy for brain metastases: secondary analysis of the JROSG 99-1 randomized clinical trial. JAMA Oncol.

[13] Sperduto PW, Chao ST, Sneed PK (2010). Diagnosis-specific prognostic factors, indexes, and treatment outcomes for patients with newly diagnosed brain metastases: a multi-institutional analysis of 4,259 patients. Int J Radiat Oncol Biol Phys.

[14] Chang EL, Wefel JS, Hess KR (2009). Neurocognition in patients with brain metastases treated with radiosurgery or radiosurgery plus whole-brain irradiation: a randomised controlled trial. Lancet Oncol.

[15] Gondi V, Pugh SL, Tome WA (2014). Preservation of memory with conformal avoidance of the hippocampal neural stem-cell compartment during whole-brain radiotherapy for brain metastases (RTOG 0933): a phase II multi-institutional trial. J Clin Oncol.

[16] Du Four S, Hong A, Chan M (2014). Symptomatic histologically proven necrosis of brain following stereotactic radiation and ipilimumab in six lesions in four melanoma patients. Case Rep Oncol Med.

[17] Lim SH, Lee JY, Lee MY (2015). A randomized phase III trial of stereotactic radiosurgery (SRS) versus observation for patients with asymptomatic cerebral oligo-metastases in non-small-cell lung cancer. Ann Oncol.

[18] Fogarty G, Morton RL, Vardy J (2011). Whole brain radiotherapy after local treatment of brain metastases in melanoma patients-a randomised phase III trial. BMC Cancer.

[19] Fogarty GB, Hong A, Dolven-Jacobsen K (2015). First interim analysis of a randomised trial of whole brain radiotherapy in melanoma brain metastases confirms high data quality. BMC Res Notes.

[20] Hamilton DW, de Salis I, Donovan JL (2013). The recruitment of patients to trials in head and neck cancer: a qualitative study of the EaStER trial of treatments for early laryngeal cancer. Eur Arch Otorhinolaryngol.

[21] Patchell RA, Tibbs PA, Regine WF (1998). Postoperative radiotherapy in the treatment of single metastases to the brain: a randomized trial. JAMA.

[22] Roos DE, Smith JG, Stephens SW (2011). Radiosurgery versus surgery, both with adjuvant whole brain radiotherapy, for solitary brain metastases: a randomised controlled trial. Clin Oncol (R Coll Radiol).

[23] Kocher M, Soffietti R, Abacioglu U (2011). Adjuvant whole-brain radiotherapy versus observation after radiosurgery or surgical resection of one to three cerebral metastases: results of the EORTC 22952-26001 study. J Clin Oncol.

